# Serum *α*-linolenic acid, not intake, associates with reduced arterial stiffness assessed by brachial-ankle pulse wave velocity: an exploratory *post hoc* analysis of a randomized controlled trial

**DOI:** 10.3389/fnut.2026.1742972

**Published:** 2026-02-18

**Authors:** Nanaka Ando, Naohisa Nosaka, Kazuhiko Kato

**Affiliations:** 1 Strategic Invention R&D, The Nisshin OilliO Group, Ltd., Yokohama, Kanagawa, Japan; 2 Kato Clinic, Komae, Japan

**Keywords:** arterial stiffness, baPWV, cardiovascular disease, lipid absorption, lipid metabolism, omega-3 fatty acids, *post hoc* analysis, *α*-Linolenic acid

## Abstract

**Introduction:**

Our previous study showed that *α*-linolenic acid (ALA) intake was associated with a reduction in brachial-ankle pulse wave velocity (baPWV) in healthy middle-aged Japanese women. However, it remains unclear whether this effect depends on the ingested ALA itself or its metabolites. This study aimed to clarify two points: first, whether there is a relationship between ALA intake and serum n-3 fatty acid concentrations; and second, whether ALA intake or serum concentrations of n-3/n-6 fatty acids are related to the previously reported effect of ALA on maintaining vascular flexibility.

**Methods:**

We conducted a *post hoc* analysis of a previously reported randomized controlled trial. Correlation analyses assessed the relationships between ALA intake and serum n-3 fatty acids. Associations of baPWV with n-3/n-6 fatty acid intake and serum concentrations were tested using an analysis of covariance-type linear model.

**Results:**

No significant correlation was found between ALA intake and serum n-3 fatty acid concentrations. Serum ALA correlated positively with serum docosapentaenoic acid (DPA) (*p* < 0.001, *r* = 0.444), docosahexaenoic acid (DHA) (*p* = 0.006, *r* = 0.292), and the sum of serum eicosapentaenoic acid, DPA, and DHA (*p* = 0.009, *r* = 0.275). ALA intake was not associated with baPWV, whereas serum ALA showed a significant negative association with baPWV (*p* = 0.027, regression coefficient: −2.45); serum arachidonic acid showed a significant positive association (*p* = 0.047, regression coefficient: 1.11). No associations were observed for other n-3/n-6 fatty acids.

**Discussion:**

This study suggests substantial inter-individual variation in the relationship between ALA intake and serum ALA concentration. Furthermore, it suggests that serum ALA concentration, rather than ALA intake, may be more closely associated with vascular stiffness, as assessed by baPWV.

**Clinical trial registration:**

https://center6.umin.ac.jp/cgi-open-bin/ctr/ctr_view.cgi?recptno=R000060078, identifier UMIN000052677.

## 1 Introduction

*α*-linolenic acid (ALA) is an n-3 essential fatty acid, primarily derived from plant sources. Among commonly used vegetable oils, ALA accounts for approximately 10% of the fatty acids in soybean and rapeseed oils, and about 50% in flaxseed oil (1). ALA reportedly has antioxidant and anti-inflammatory properties (2–4). Furthermore, ALA intake and blood concentrations are associated with reduced risks of cardiovascular disease, coronary heart disease, and all-cause mortality (5). Our previous study confirmed that ALA intake reduced brachial-ankle pulse wave velocity (baPWV) (6). baPWV is an indicator of vascular stiffness (lower values indicate greater arterial compliance), and higher values are reportedly associated with an increased risk of cardiovascular disease (7).

ALA is converted to eicosapentaenoic acid (EPA) and docosahexaenoic acid (DHA) via enzymatic reactions involving desaturases and elongases, primarily in the liver (8). The conversion rates of ALA to EPA and DHA are reportedly approximately 8–12% and less than 1%, respectively (9, 10). These conversion rates vary with age and sex (11). Furthermore, the metabolic pathways of n-3 fatty acids originating from ALA and those of n-6 fatty acids originating from linoleic acid (LA) are mutually competitive (12). LA is converted to *γ*-linolenic acid (GLA), dihomo-γ-linolenic acid (DGLA), and arachidonic acid (AA) via enzymatic reactions (12). Both n-3 and n-6 fatty acids serve as precursors for eicosanoids, biologically active lipid mediators that can antagonistically regulate inflammatory responses (13).

It remains unclear whether the beneficial effect of ALA on vascular stiffness reported in our previous study depends on the ingested ALA itself, the n-3 fatty acids produced by its conversion, or its metabolites. Lipid energy metabolism, fat accumulation, and eicosanoid profiles are known to be influenced by various factors such as genes, circadian rhythms, and dietary patterns (14–16), suggesting substantial inter-individual variability. It has been reported that the metabolism and bioavailability of ALA and n-3 fatty acids are influenced by genetic variants, gut microbiome, and metabolic phenotypes (17–19). Therefore, even when consuming the same amounts of ALA and n-3 fatty acids, health effects may differ between individuals due to these characteristics. However, the relationship between the vascular effect of ALA intake and inter-individual variability has not been fully understood. In this study, we conducted a *post hoc* analysis with two objectives, using only data from participants who were allocated to the ALA intervention groups in a previously reported randomized controlled trial: first, to clarify whether there is a relationship between ALA intake and serum n-3 fatty acid concentrations; and second, to clarify whether ALA intake or serum concentrations of various n-3 and n-6 fatty acids contribute to the previously reported effect of ALA on vascular stiffness.

## 2 Materials and methods

### 2.1 Data collection

We conducted a *post hoc* analysis of a previously reported randomized, double-blind, parallel-group placebo-controlled trial (6). It was conducted between January and April 2024 in healthy, middle-aged Japanese women aged 45–65 years. Individuals with a current or past serious disease requiring medication or ongoing medical treatment, those with irregular lifestyles (e.g., night shift work), and those who regularly used health foods, supplements, or medicines that affect antioxidant capacity were excluded. Each participant consumed either a control food or one of three test foods containing different doses of ALA for 12 weeks. The details of the participant inclusion and exclusion criteria, sample size determination, requirements during the intervention and on assessment days, and surveys and measurement methods were identical to the details described in our previous report. The trial was conducted by the contract research organization HUMA R&D at AMC Shinjuku Clinic and Shinjuku Research Center of M&I Science Co., Ltd., under physician’s supervision. The study adhered to the principles of the Declaration of Helsinki (20) and was approved by the Miyake Clinic Institutional Review Board (approval number: RD12013AS04, approval date: November 28, 2023). The study was registered with the UMIN-CTR before commencement (UMIN-ID: UMIN000052677). Randomization was performed by a third party not involved in the study. The allocation manager maintained the strict confidentiality of the allocation list until unblinding to maintain blinding among all other study personnel.

In our previously reported study, we analyzed data from a total of 120 participants in the ALA test groups and the control group to evaluate the effects of ALA intake on measured parameters. In this *post hoc* analysis, to explore inter-individual variability in the effects among participants who consumed ALA, the control group was excluded from the analytical sample. Consequently, the study is no longer a randomized controlled trial but rather an observational analysis of associations. One participant who withdrew during the trial period was excluded, resulting in data from 89 participants being used. There were three intervention groups. The daily ALA content of the test food and the number of participants in each group were as follows: low-dose group (0.97 g/day, 30 participants), medium-dose group (1.36 g/day, 29 participants), and high-dose group (2.13 g/day, 30 participants). Details regarding the composition and preparation of the test food can be found in our previous report (6). In this study, we used data on ALA intake, serum fatty acid concentrations, baPWV, and body weight measured at baseline and after 12 weeks.

ALA intake was calculated by summing the fatty acid content of the test food with each participant’s daily dietary ALA intake, as assessed using the brief-type self-administered diet history questionnaire (BDHQ) (21).

Serum fatty acid concentrations were measured as previously described (22): serum samples were hydrolyzed and methyl-esterified and then analyzed by gas chromatography.

baPWV was measured using the HBP-8000 (OMRON Corporation) (23). Following previous studies (24, 25), the mean of the left and right baPWV values was used for the statistical analysis.

### 2.2 Statistical analysis

In this study, we conducted a *post hoc* analysis rather than the analyses prespecified during the clinical trial phase.

For ALA intake and serum n-3 fatty acid concentrations, data normality was assessed using the Shapiro–Wilk test. If normality was met, Pearson’s correlation coefficient (*r*) was calculated between ALA intake and serum n-3 fatty acid concentrations; otherwise, Spearman’s rank correlation coefficient (*ρ*) was used. Analyses were performed for both the actual values and the change values (values at 12 weeks minus baseline values).

The relationship between baPWV and n-3/n-6 fatty acid intake and serum concentrations was analyzed using an analysis of covariance (ANCOVA)-type linear model. The objective variable was baPWV. The explanatory variables were the intake and serum concentrations of n-3 and n-6 fatty acids (ALA, EPA, docosapentaenoic acid (DPA), DHA, LA, GLA, DGLA, and AA), the serum EPA:AA ratio, and the n-6:n-3 ratio. The covariates were the baseline baPWV, baseline values of the explanatory variables, age, BMI, and habitual n-3 and n-6 fatty acid intake prior to the intervention. We evaluated the extent to which each explanatory variable explained the variation in baPWV. Statistical significance was determined by the *p*-value for each explanatory variable, and the effect size was assessed by the corresponding regression coefficient.

## 3 Results

Table 1 shows the baseline clinical data of the participants.

**Table 1:** Baseline clinical characteristics of the analyzed participants (*n* = 89)^1^.

Characteristics	Values	Low-dose group	Medium-dose group	High-dose group
Age	years	54.6 ± 5.2	53.8 ± 5.8	54.4 ± 5.2
Height	cm	157.9 ± 6.0	159.1 ± 3.6	158.3 ± 5.6
Body weight	kg	58.0 ± 6.3	58.3 ± 4.9	59.8 ± 7.0
BMI	kg/m^2^	23.2 ± 1.6	23.0 ± 1.8	23.7 ± 1.8
SBP	mmHg	111.7 ± 11.3	111.8 ± 10.5	117.7 ± 15.0
DBP	mmHg	74.8 ± 8.0	74.8 ± 7.8	79.8 ± 9.0
baPWV	cm/s	1325.4 ± 171.8	1349.1 ± 183.0	1391.6 ± 229.7
ALA in blood serum	μg/mL	22.2 ± 14.8	20.2 ± 10.0	21.8 ± 10.2
ALA intake (without test food)	mg/day	1489.5 ± 576.7	1404.4 ± 505.9	1522.5 ± 430.2

1 Values are given as mean ± standard deviation. BMI, body mass index.

The results of the correlation analysis between ALA intake and serum n-3 fatty acid concentrations are presented in Table 2. No significant correlation was observed between ALA intake and serum n-3 fatty acid concentrations in either the actual values or the change values. For the actual values, serum ALA concentration showed a significant positive correlation with serum DPA concentration (*p* < 0.001, *r* = 0.444) and a weak but significant positive correlation with serum DHA concentration (*p* = 0.006, *r* = 0.292) as well as with the sum of the concentrations of serum EPA, DPA, and DHA (*p* = 0.009, *r* = 0.275). For the change values, serum ALA concentration showed a weak but significant positive correlation with serum DPA concentration (*p* = 0.004, *r* = 0.301).

**Table 2:** Correlation between ALA intake and serum n-3 fatty acid concentrations.

Outcome	Values	Actual value	Change value	Correlation with ALA intake (*r*, *p*)		Correlation with ALA in blood serum (*r*, *p*)	
				Actual value	Change value	Actual value	Change value
ALA intake	mg/day	2941.32 ± 704.91	1468.42 ± 500.97	–	–	–	–
ALA in blood serum (18:3n-3)	μg/mL	27.94 ± 14.14	6.56 ± 12.22	0.115 (0.284)	0.068 (0.524)	–	–
EPA in blood serum (20:5n-3)	μg/mL	46.24 ± 27.18	3.35 ± 24.42	0.176 (0.099)	0.074 (0.493)	0.151 (0.157)	0.032 (0.768)
DPA in blood serum (22:5n-3)	μg/mL	18.83 ± 5.85	−0.54 ± 4.33	−0.026 (0.812)	−0.208 (0.051)	0.444 (< 0.001*)	0.301 (0.004*)
DHA in blood serum (22:6n-3)	μg/mL	98.92 ± 31.33	−5.04 ± 20.11	0.116 (0.278)	0.099 (0.358)	0.292 (0.006*)	0.020 (0.849)
Total EPA + DPA + DHA in blood serum	μg/mL	164.00 ± 58.3	−2.22 ± 42.90	0.108 (0.313)	0.099 (0.354)	0.275 (0.009*)	0.070 (0.516)
EPA:AA ratio		0.26 ± 0.15	0.03 ± 0.14	0.106 (0.322)	−0.013 (0.903)	0.128 (0.230)	−0.015 (0.892)
n-6:n-3 ratio		6.92 ± 2.23	−0.01 ± 1.75	−0.179 (0.093)	−0.078 (0.467)	−0.101 (0.349)	0.018 (0.869)

1 Values are given as mean ± standard deviation. * *p* < 0.05 (Pearson’s r when normality was met; otherwise Spearman’s ρ). ALA, α-linolenic acid; EPA, eicosapentaenoic acid; DPA, docosapentaenoic acid; DHA, docosahexaenoic acid; AA, arachidonic acid.

The results of the ANCOVA-type linear model analysis are shown in Table 3. The *p*-value for ALA intake as an explanatory variable was 0.797, indicating no significant association with baPWV. No significant association with baPWV was observed for the intake of other n-3 and n-6 fatty acids. The *p*-value for serum ALA concentration as an explanatory variable was 0.027, showing a significant negative association with baPWV (regression coefficient: −2.45). The *p*-value for serum AA concentration as an explanatory variable was 0.047, showing a significant positive association (regression coefficient: 1.11). Serum concentrations of other n-3 and n-6 fatty acids showed no significant association with baPWV. The results of the model diagnostics and multicollinearity assessment are presented in Supplementary Table 1.

**Table 3:** The relationship between baPWV and n-3/n-6 fatty acid intake and serum concentrations.

Outcome domain	Category	Fatty acid species	Regression coefficient	*p*-value	95% CI
Intake amount	n-3 fatty acids	ALA (18:3)	−0.01	0.797	−0.06, 0.05
		EPA (20:5)	−0.28	0.216	−0.73, 0.17
		DPA (22:5)	−0.94	0.229	−2.48, 0.60
		DHA (22:6)	−0.20	0.181	−0.49, 0.09
	n-6 fatty acids	LA (18:2)	−0.02	0.141	−0.05, 0.01
		GLA (18:3)	−0.14	0.985	−14.19, 13.92
		DGLA (20:3)	−4.15	0.446	−14.93, 6.63
		AA (20:4)	−0.63	0.447	−2.29, 1.02
Blood concentration	n-3 fatty acids	ALA (18:3)	−2.45	0.027*	−4.61, −0.29
		EPA (20:5)	−0.34	0.557	−1.50, 0.82
		DPA (22:5)	−3.49	0.269	−9.73, 2.75
		DHA (22:6)	−0.39	0.567	−1.72, 0.95
	n-6 fatty acids	LA (18:2)	−0.02	0.825	−0.20, 0.16
		GLA (18:3)	0.50	0.881	−6.05, 7.04
		DGLA (20:3)	−0.24	0.890	−3.67, 3.19
		AA (20:4)	1.11	0.047*	0.01, 2.20
	EPA:AA ratio		−125.00	0.215	−324.17, 74.16
	n-6:n-3 ratio		13.86	0.083	−1.87, 29.59

* *p* < 0.05 (analysis of covariance (ANCOVA)-type linear model). CI, confidence interval; LA, linoleic acid; GLA, γ-linolenic acid; DGLA, dihomo-γ-linolenic acid.

**Figure 1:**
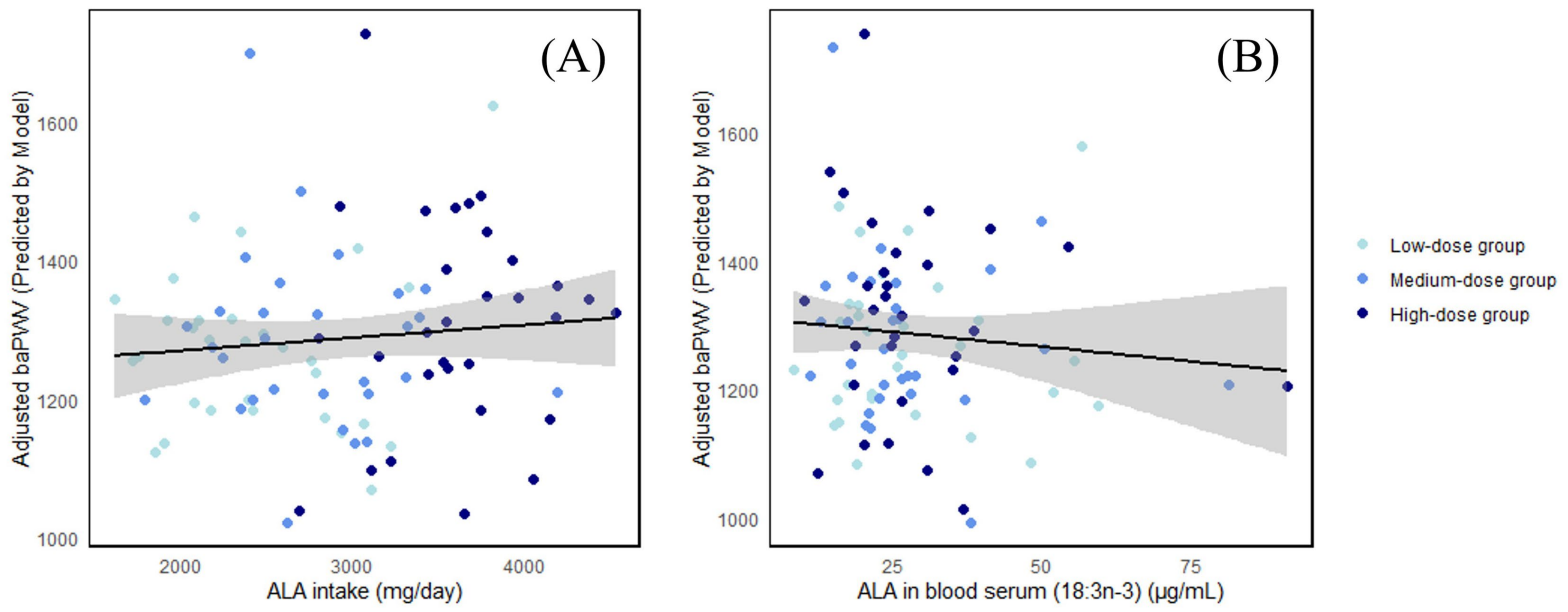
**(A)** The relationship between ALA intake and baPWV; **(B)** The relationship between serum ALA concentration and baPWV.

The relationships among ALA intake, serum ALA concentration, and baPWV are shown in Figure 1.

## 4 Discussion

Most dietary fatty acids are triglycerides (26). Ingested triglycerides are first broken down into free fatty acids and 2-monoacylglycerols by lingual and gastric lipases, and subsequently by pancreatic lipase. Hydrolyzed products (long-chain fatty acids and monoacylglycerols) form micelles stabilized by bile salts. As these micelles pass through the small intestine, fatty acids and monoacylglycerols are released, absorbed by enterocytes, and re-esterified into triglycerides. These triglycerides are assembled into chylomicrons and transported throughout the body via the lymphatic system. Meanwhile, medium-chain fatty acids are transported directly into the hepatic portal vein and delivered to the liver (27). Absorbed triglycerides are used for energy, stored as subcutaneous fat, incorporated into cell membranes, or used to act as precursors for lipid mediators, such as eicosanoids (28, 29).

Lipid energy metabolism, fat accumulation, and eicosanoid profiles are influenced by various factors, such as genes, circadian rhythms, and dietary patterns (14–16), suggesting substantial inter-individual variation in the fate of ingested lipids. Furthermore, for food-derived bioactive components, the magnitude of the effect often varies across individuals, with reports of responders who readily exhibit effects and non-responders who do not (30). In addition, for n-3 fatty acids, not only their intrinsic effects, but also gut microbiota-derived metabolites and oxylipins are reportedly associated with health outcomes (19, 31). The postprandial and longer-term kinetics after ALA intake, as well as the detailed mechanisms underlying the health effects, are not fully understood.

Our previous report demonstrated the average effect of the intervention at the group level through between-group comparisons (6), whereas the present study focused on the intervention groups and evaluated relationships with baPWV by examining individual ALA intake and serum concentrations of n-3 and n-6 fatty acids.

Among blood lipids, chylomicrons reflect short-term postprandial intake, blood triglycerides reflect intake over the past several hours to days, and cholesteryl esters and phospholipids reflect dietary intake over several days (32). In this trial, as per the study schedule, serum for total lipid analysis was collected at least 12 h after the test food’s final intake. Therefore, the serum fatty acid profile in this study likely reflects continuous intake over several days or longer, rather than the immediate effect of a single intake of the test food. Some studies have reported a significant relationship between ALA intake and serum ALA concentrations, whereas others have not (33–36). Furthermore, some reports indicate that the relationship between intake and blood concentration is weaker for ALA than for DHA (34). The fact that ALA intake does not necessarily correlate with blood concentration may be partly influenced by the conversion of ALA to EPA and DHA.

In this study, ALA intake did not show a significant correlation with serum ALA concentration. In our previous report, serum ALA concentrations were significantly higher in all three ALA-dose groups than in the control group in between-group comparisons and were significantly higher at 12 weeks than at baseline in within-group comparisons (6). Furthermore, in the high-dose group, serum EPA concentration also showed a significant increase compared with the control group in both between-group and within-group comparisons. Thus, while ALA intake increased serum ALA concentrations at the group level, scatter plots of individual ALA intake versus serum ALA concentration revealed no significant relationship. These results suggest substantial inter-individual variability in the relationship between ALA intake and serum ALA concentration. Furthermore, while no significant correlation was observed between ALA intake and serum concentrations of ALA metabolites (EPA, DPA, and DHA), a significant positive correlation was found between serum ALA concentration and serum concentrations of ALA metabolites (EPA, DPA, and DHA). The rate of ALA appearance in blood and its conversion rates to EPA, DPA, and DHA could not be clarified in this trial; verification using ^13^C-labeled ALA intake is needed.

In our previous report, baPWV values were significantly lower than those in the control group in all three ALA-dose groups and were significantly lower at 12 weeks than at baseline (6). However, when analyzing the relationship between ALA intake and baPWV, specifically in participants in the intervention groups from the same randomized, double-blind, parallel-group placebo-controlled trial, no significant association was found between the two variables. Thus, while ALA intake was associated with lower baPWV at the group level, no individual-level dose–response relationship was observed. Regarding serum n-3 fatty acid concentrations, serum ALA concentration showed a significant association with baPWV: higher serum ALA levels were associated with lower baPWV values. In this *post hoc* analysis, the association with baPWV was more evident for serum ALA concentrations than for ALA intake; however, mediators, confounders, and colliders have not been identified. No significant association was observed between serum EPA, DPA, or DHA concentrations and baPWV. Furthermore, no significant association was observed between the intake of EPA, DPA, or DHA and baPWV. This raises the possibility that ALA itself—rather than the n-3 fatty acids converted from ALA *in vivo*—may contribute to the maintenance of vascular stiffness. ALA is known to be less prone to oxidation than EPA and DHA (37). Clarifying the health benefits of ALA itself is useful for overcoming the disadvantages of EPA and DHA in food processing and preservation and for efficiently providing the health benefits of n-3 fatty acids. Notably, prior studies have reported that higher ALA intake and blood concentrations are associated with a reduced risk of cardiovascular disease and coronary heart disease (5).

The EPA:AA ratio has been noted for its clinical utility in cardiovascular disease, with lower EPA:AA ratios reportedly associated with an increased risk of cardiovascular disease (38, 39). In this study, no significant association was found between the EPA:AA ratio and baPWV, but serum AA alone showed a significant positive association with baPWV. The metabolism of ALA and AA is known to be mutually competitive (12). The negative association between baPWV and serum ALA, and the positive association with serum AA, are consistent with findings from previous studies. No significant association was observed between baPWV and the intake or serum concentrations of LA, GLA, or DGLA, which are precursors of AA. Atherosclerosis is reportedly associated with inflammation (40). Furthermore, AA is a precursor of pro-inflammatory eicosanoids (13). Although eicosanoids were not measured in this study and causality cannot be established, the positive association observed between serum AA concentration and baPWV could plausibly be related to pro-inflammatory eicosanoids.

Based on this *post hoc* analysis, for each 1 μg/mL increase in serum ALA concentration, the estimated decrease in baPWV was approximately 2.45 cm/s. In this study, the change in serum ALA among participants in the ALA groups was 6.56 ± 12.22 μg/mL (6). Prior studies evaluating the effects of pharmacotherapy, exercise therapy, or dietary interventions on baPWV have reported intervention-induced reductions of roughly 50–200 cm/s (41–44). Although the estimated change observed in our study is smaller than those reported previously, it was statistically significant, suggesting that higher serum ALA concentrations are associated with lower baPWV and may be of some relevance. In addition, the partial R^2^ (as the effect size) for serum ALA concentration was 0.03 in our analysis. While definitions of effect size vary by research area and analytic approach, it has been suggested that, in multiple regression, R^2^ values of 0.02, 0.13, and 0.26 can be interpreted as “small,” “medium,” and “large,” respectively (45). It has also been noted that, even when the theoretical effect is “medium,” studies with substantial noise may yield numerically “small” effect sizes (45). Thus, although the partial R^2^ for serum ALA concentration is small—indicating that the proportion of variance explained by this predictor is limited—we consider it to have some relevance in explaining baPWV.

Serum ALA concentration showed a significant negative association with baPWV, but ALA intake showed no significant association with baPWV. Prior work has shown that, whereas associations between n-3 fatty acid intake and venous thromboembolism risk have been inconsistent, serum n-3 fatty acid concentrations are inversely associated with that risk (46). These observations further suggest that individuals with low serum ALA levels (reflecting lower bioavailability or incorporation) may require higher ALA intake.

This study has several limitations. First, in addition to being a *post hoc* analysis rather than one prespecified during the clinical trial phase, the use of measurements from intervention groups only renders the analysis observational, thereby limiting causal interpretation. Second, we assessed only baPWV, serum lipids, and dietary surveys by BDHQ; thus, we were unable to evaluate factors that may contribute to inter-individual variability, such as dietary patterns, genetic influences, and metabolic phenotypes. Moreover, because measurements were obtained at only two time points (pre- and post-intervention), intra-individual variability could not be assessed. These constraints limit the interpretation of the findings. Third, the study was conducted in a population of healthy middle-aged Japanese women, so the generalizability of the results to populations of different ages, sexes, and ethnicities is unclear. In addition, the sample size was 120, which may be limiting. Fourth, while serum fatty acid concentrations were measured, fatty acid concentrations in fractions, such as red blood cell membranes and phospholipids, as well as fatty acid metabolites, were not measured, and their influences remain unclear. Fifth, although the relationship between intake and serum concentrations of ALA and other n-3 fatty acids was analyzed, the conversion rate of ALA was not measured. In addition, this study was conducted by employees of a company involved in the manufacture and sale of vegetable oils. To mitigate potential bias, the clinical trial was carried out by an independent contract research organization according to prespecified procedures under blinding. Nevertheless, potential biases related to conflicts of interest cannot be completely ruled out.

Despite several limitations, its results suggest that serum ALA concentration—rather than ALA intake—may be more clearly associated with baPWV and with serum EPA, DPA, and DHA concentrations. It also indicates substantial inter-individual variability in the relationship between ALA intake and serum ALA concentration. Future research should include data collection from participants of different ages and sexes, measurement of fraction-specific fatty acid composition (e.g., red blood cell membranes and phospholipids), and estimation of ALA conversion rates using ^13^C-labeled ALA. Furthermore, studies incorporating individual genetic information and gut microbiome data are needed to elucidate individual differences in ALA absorption, metabolism, and health effects.

## Data Availability

The raw data supporting the conclusions of this article will be made available by the authors, without undue reservation. Requests to access these datasets should be directed to Nanaka Ando, n-andoh@nisshin-oillio.com.
