# Supplementary material for: Serum α-linolenic acid, not intake, associates with reduced arterial stiffness assessed by brachial-ankle pulse wave velocity: an exploratory post hoc analysis of a randomized controlled trial
Source: Front Nutr. 2026 Feb 18;13:1742972. doi: 10.3389/fnut.2026.1742972 (PMC12958356; doi:10.3389/fnut.2026.1742972)
Supplement: Supplementary file 1 [file Table_1.docx]

**Supplementary Table 1. Model diagnostics, multicollinearity assessment, and explained variance.**

|  |  |  | **model diagnostics** | | | **Max VIF value** | **explained variance of explanatory variable** |
| --- | --- | --- | --- | --- | --- | --- | --- |
|  |  |  | ***p*-value of**  **normality of residual** | ***p*-value of heteroscedasticity** | **model fit** |  |  |
| Intake amount | n-3 fatty acids | ALA (18:3) | 0.597 | 0.149 | 0.570 | 19.4 | 0.007 |
|  |  | EPA (20:5) | 0.804 | 0.193 | 0.577 | 25.9 | 0.009 |
|  |  | DPA (22:5) | 0.692 | 0.180 | 0.578 | 47.6 | 0.011 |
|  |  | DHA (22:6) | 0.612 | 0.193 | 0.580 | 38.0 | 0.010 |
|  | n-6 fatty acids | LA (18:2) | 0.690 | 0.123 | 0.583 | 4403.2 | 0.012 |
|  |  | GLA (18:3) | 0.639 | 0.245 | 0.569 | 4.87 | 0.004 |
|  |  | DGLA (20:3) | 0.705 | 0.151 | 0.575 | 18.8 | 0.003 |
|  |  | AA (20:4) | 0.714 | 0.136 | 0.580 | 17.0 | 0.005 |
| Blood concentration | n-3 fatty acids | ALA (18:3) | 0.815 | 0.285 | 0.600 | 4.09 | 0.030 |
|  |  | EPA (20:5) | 0.429 | 0.136 | 0.590 | 4.30 | 0.015 |
|  |  | DPA (22:5) | 0.846 | 0.140 | 0.579 | 4.33 | 0.019 |
|  |  | DHA (22:6) | 0.627 | 0.153 | 0.570 | 4.44 | 0.013 |
|  | n-6 fatty acids | LA (18:2) | 0.824 | 0.280 | 0.570 | 4.19 | 0.005 |
|  |  | GLA (18:3) | 0.720 | 0.007 | 0.569 | 4.09 | 0.032 |
|  |  | DGLA (20:3) | 0.672 | 0.017 | 0.572 | 4.19 | 0.011 |
|  |  | AA (20:4) | 0.781 | 0.088 | 0.603 | 4.19 | 0.092 |
|  | EPA:AA ratio |  | 0.492 | 0.246 | 0.587 | 4.25 | 0.006 |
|  | n-6:n-3 ratio |  | 0.619 | 0.283 | 0.592 | 4.25 | 0.013 |

VIF, variance inflation factor; CI, confidence interval; LA, linoleic acid; GLA, γ-linolenic acid; DGLA, dihomo-γ-linolenic acid.
